# Precipitation Polymerization: A Powerful Tool for Preparation of Uniform Polymer Particles

**DOI:** 10.3390/polym14091851

**Published:** 2022-04-30

**Authors:** Randi Zhang, Rong Gao, Qingqiang Gou, Jingjing Lai, Xinyang Li

**Affiliations:** Department of Polyethylene, SINOPEC (Beijing) Research Institute of Chemical Industry Co., Ltd., Beijing 100013, China; gaor.bjhy@sinopec.com (R.G.); gouqq.bjhy@sinopec.com (Q.G.); laijj.bjhy@sinopec.com (J.L.); lixinyang.bjhy@sinopec.com (X.L.)

**Keywords:** precipitation polymerization, polymer microspheres, applications

## Abstract

Precipitation polymerization (PP) is a powerful tool to prepare various types of uniform polymer particles owing to its outstanding advantages of easy operation and the absence of any surfactant. Several PP approaches have been developed up to now, including traditional thermo-induced precipitation polymerization (TRPP), distillation precipitation polymerization (DPP), reflux precipitation polymerization (RPP), photoinduced precipitation polymerization (PPP), solvothermal precipitation polymerization (SPP), controlled/‘‘living’’ radical precipitation polymerization (CRPP) and self-stabilized precipitation polymerization (2SPP). In this review, a general introduction to the categories, mechanisms, and applications of precipitation polymerization and the recent developments are presented, proving that PP has great potential to become one of the most attractive polymerization techniques in materials science and bio-medical areas.

## 1. Introduction

Functional materials are the core of the new materials field and have been developed very rapidly in recent years [1]. The polymer microspheres with optimized characteristics, such as uniform size and shapes, the functionality of the base polymer, the morphology of the polymer particles, and the degree of crosslinking, are attractive for a wide number of applications, including supporting phases for separation science [2,3], biomedical devices [4], chromatography separation [5,6,7], casting additives [8], and controlled release reservoirs [9,10]. 

Polymer microspheres of a particular size and uniformity are generally obtained with one of the heterogeneous polymerizations, including suspension, emulsion, dispersion, and seeded polymerizations. All the methods mentioned above require the presence of a suitable stabilizer or surfactant, usually in large quantities, to induce the formation of particles and prevent the aggregation of the colloidal particles. For instance, in dispersion polymerization, polymeric particles form and precipitate from the reaction medium, and stabilizers such as poly(vinyl pyrrolidone) must be utilized to stabilize polymeric particles. Precipitation polymerization (PP), first reported by Stöver et al. [11], was a very impressive method for the preparation of surface-clean microspheres without adding any stabilizer/surfactant or other additives, in which polymeric microspheres form by self-crosslinking of monomers/crosslinkers and precipitate out of the homogeneous solution due to the newly formed polymers with low solubility in the selected reaction media (Θ solvent) [12].

**Figure 1:**
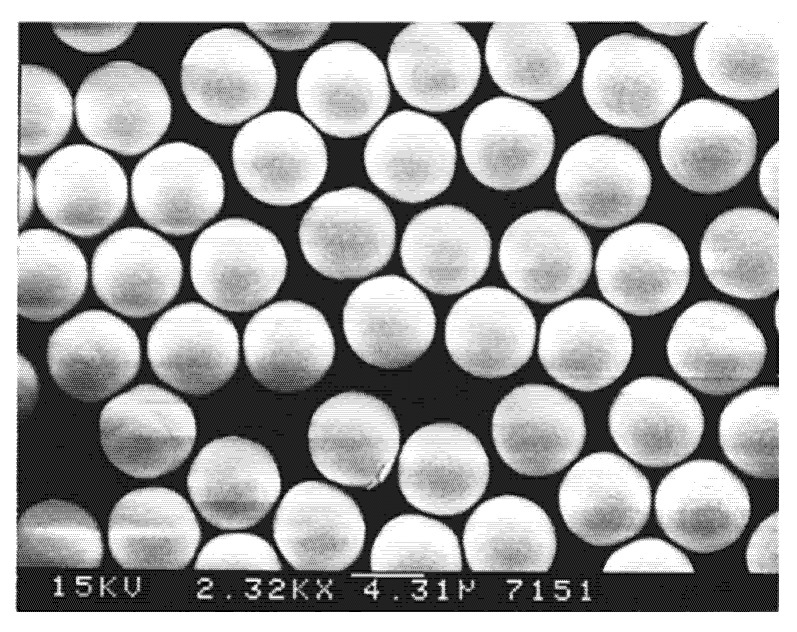
SEM image of poly(DVB-55) microspheres with porous shells prepared in a toluene/acetonitrile (40/60) mixture. Reprinted with permission from Ref. [16]. Copyright 2000 American Chemical Society.

PP starts from a homogeneous mixture of monomer, initiator, and optional solvent [11,13,14]. Stöver et al. proposed that precipitation polymerization of divinylbenzene (DVB) in near-Θ solvents is an entropic precipitation in cases where cross-linking prevents the polymer and solvent from freely mixing [15], and they further proposed the transient solvent-swollen gel layer mechanism to explain the formation, stabilization, and growth of microspheres [13,14]. Uniform spherical polymer particles are formed through the particle nucleation and growth stages, where particle nuclei are generated by the aggregation of oligomers at the beginning of the polymerization, and their growth occurs mainly through capturing oligomeric radicals from the reaction medium by their reacting with the residual vinyl groups on the surfaces of the existing particles [14]. Further examinations showed that the presence of bifunctional monomers, suitable reaction medium, and moderate shaking are the keys to the formation of highly cross-linked monodisperse microspheres and various functionalized, porous, and core-shell microspheres by PP have been well prepared [16,17]. Figure 1 shows the polydivinylbenzene (PDVB) microspheres with porous shells prepared in a toluene/acetonitrile mixture. 

**Figure 2:**
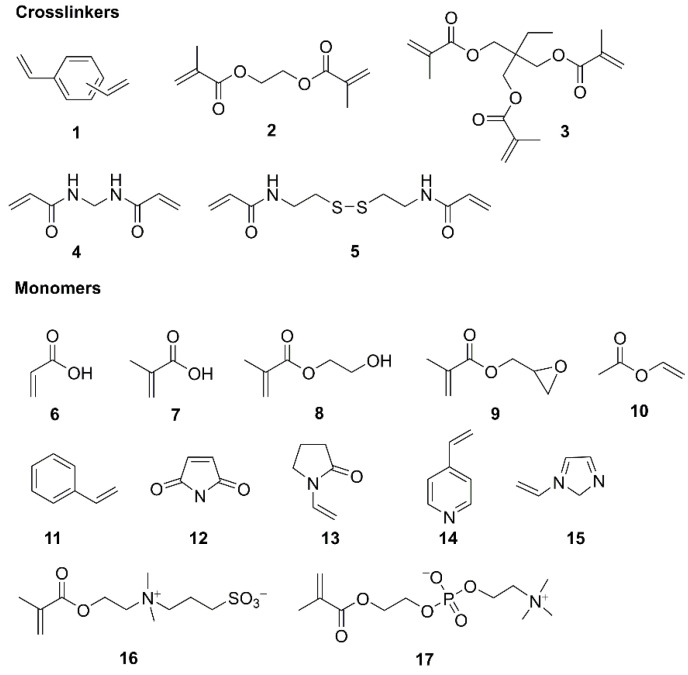
The chemical structures of crosslinkers (**1**–**5**) and monomers (**6**–**17**) for the precipitation polymerization. (**1**) Divinylbenzene, (**2**) ethylene glycol dimethylacrylate, (**3**) trihydroxymethyl propane trimethyl acrylate, (**4**) *N*,*N*′-methylenebis(acrylamide), (**5**) *N*,*N*′-Bis(acryloyl)cystamine, (**6**) acrylic acid, (**7**) methacrylic acid, (**8**) 2-hydroxyethyl methacrylate, (**9**) glycidyl methacrylate, (**10**) vinyl acetate, (**11**) styrene, (**12**) maleic anhydride, (**13**) 1-vinyl-2-pyrrolidone, (**14**) 4-vinyl pyridine, (**15**) 1-vinylimidazole, (**16**) 2-(methacryloyloxy)ethyl)-dimethyl-(3-sulfopropyl) ammonium, (**17**) 2-methacryloyloxyethyl phosphorylcholine.

Recent years have witnessed the rapid development of precipitation polymerization, and again, the method could offer an efficient way to tune the size and porosity of the spherical particles through control of the polymerization condition, as well as avoiding environmental pollution and increasing costs of additives, thus opening up numerous potential applications of functional materials [6,18]. More researchesr have been focused on such techniques, such as molecularly imprinted polymers (MIPs) [19,20,21,22,23] or liquid crystalline (LC) polymer particles [24,25], which have been attractive in recent years. Figure 2 shows the typical crosslinkers and monomers (including copolymer monomers) adopted for PP, along with 2,2-azobisisobutyronitrile (AIBN) as the frequently used initiator.

## 2. Categories of Precipitation Polymerization

**Figure 3:**
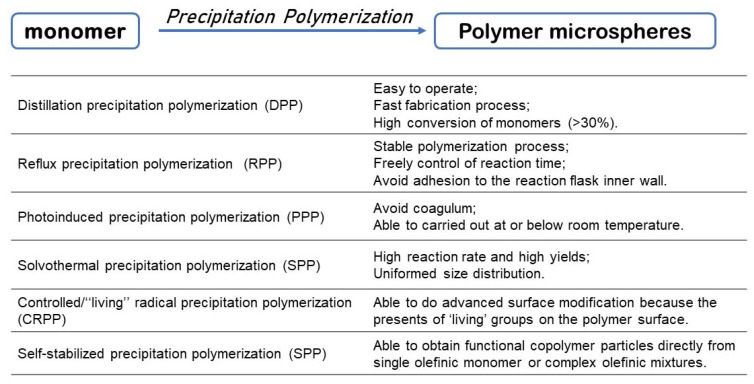
Advantages of different precipitation polymerization techniques in preparation of polymer microspheres [29,30,31,32,33,34,35,36,37,38,39].

During the development of PP, several different kinds of approaches based on the conventional free radical polymerization mechanism have been developed for the preparation of uniform crosslinked spherical polymer particles. Except for traditional thermo-induced precipitation polymerization (TRPP) [16,17,26,27,28], new methods such as distillation precipitation polymerization (DPP) [29], reflux precipitation polymerization (RPP) [30,31], photoinduced precipitation polymerization (PPP) [32,33,34], solvothermal precipitation polymerization (SPP) [35], controlled/‘‘living’’ radical precipitation polymerization (CRPP) [36,37] and self-stabilized precipitation polymerization (2SPP) [38,39] have also been successively developed (Figure 3). So far, in addition to DVB, many other multifunctional monomers have been applied to prepare crosslinked polymeric microspheres, including methacrylates, acrylamides, styrene, acrylonitrile, and *p*-chloromethylstyrene. Functionalization of polymer particles is of great importance for their application, and microspheres have unreacted double bonds on their surface, allowing their post functionalization and the synthesis of microspheres with core-shell structures [40,41].

Over the years, many research groups have become interested in further exploring the scope, versatility, and mechanisms of PP, including addressing the challenges that remain in the enhancement of productivity and solving the restriction of solvents in order to enlarge more types of monomers [42,43,44]. In addition to being a source of academic interest, those new systems and their resulting crosslinked polymeric microspheres have displayed genuine potential for industrial applications and have been systematically summarized [12]. Herein, we collect together some of the more recent results within the field, with the main emphasis being placed on the new types of precipitation polymerization, such as categories, origin, and mechanisms. We hope our collected findings will illustrate the value of the research and, furthermore, will encourage other researchers to become involved in the challenges ahead.

### 2.1. Distillation/Reflux Precipitation Polymerization 

Distillation precipitation polymerization (DPP) is a method of controlling microsphere formation by solvent distillation during the polymerization process. The solvent will evaporate out with the increase in the temperature, and the concentration of the reactants gradually increases, which would result in faster reaction rates [45]. Huang and Yang et al. have made efforts to achieve the hydrophilic property of the surface of the microspheres with wider ranges of polarity and functionality [29], as well as giving the growth mechanism for leading to good spherical shape [46,47]. During the investigation of the hydrophilic poly(methacrylic acid) (PMAA) polymer particles, they proposed that the presence of hydrogen bonding interactions between the carboxylic acid groups as a non-covalent linkage or physical crosslinking plays a key role in the nucleation process [48]. Functional monomers such as methacrylates, acrylates, chloromethylstyrene, amide, pyrrolidone, carboxylic acid, and acrylonitrile were introduced to prepare narrow-disperse microspheres [49,50,51,52,53,54]; meanwhile, the tri- or tetra-layer polymer and inorganic/polymer composite/hybrid microspheres with movable inner core have also achieved successfully [55,56]. Wang et al. then proposed a more efficient approach, reflux precipitation polymerization (RPP) [31], by simplifying the apparatus to a handy manual refluxing operation and introducing mechanical stirring, in which the well-defined polymeric nanogels could precipitate from the poor solvent without adhesion to the reaction flask inner wall that makes it easily be scaled up and freely time-controlled [30]. Currently, most of the efforts have focused on the combination of DPP or RPP with other polymerization techniques (e.g., living radical polymerization [57,58] or ‘click’ chemistry [59,60,61]) to meet the use of specific materials in various areas, such as drug/gene delivery, glycopeptides/protein enrichment [62,63], molecular recognition [45] and cell detection. For the precisely controlled structures, modular functionalities, and desirable payload encapsulation [49,64,65,66,67,68], we can foresee its huge potential in preparing novel multifunctional nanostructures to meet the requirements of multidisciplinary applications [69].

**Figure 4:**
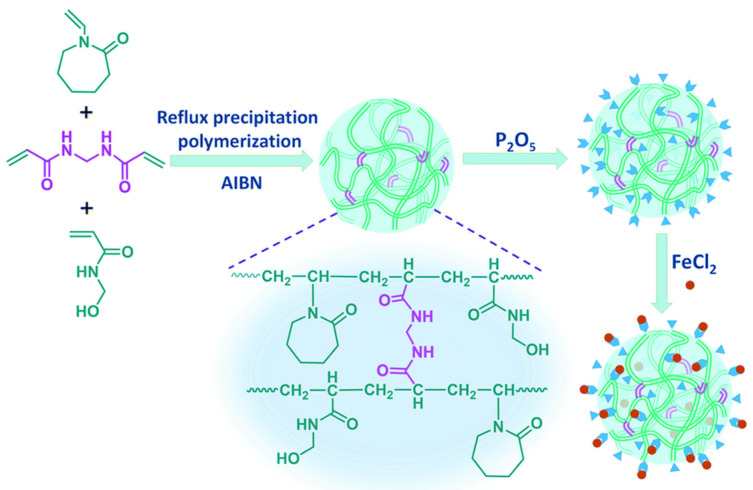
The synthetic scheme of the iron nanozymes based on poly(*N*-vinyl caprolactam) (PVCL). Reprinted with permission from Ref. [76]. Copyright 2020 Royal Society of Chemistry.

In particular, a series of biodegradable zwitterionic stimuli-responsive (e.g., pH/redox) nanogels with crosslinker that contains disulfide bond or metal ions, especially for drug delivery, via either DPP/RPP or aqueous precipitation polymerization, have been prepared for its advantages of avoiding the application of toxic surfactants and stabilizers [64,65,66,67,68,70,71,72,73,74,75,76]. Much of the residual functional groups on the core surface, which enables the polymer nanoparticles for further modification, remained [77,78]. The applications in recognizing small organic molecules, especially for MIPs [79,80], or facilitating the uniform polymer coating on the magnetic nanoparticle surface [81], have also attracted much attention. Zhao et al. combined RAFT and RPP for the synthesis of novel water-compatible MIPs with improved mass transport [57]. Jiang et al. prepared a novel kind of Ni^2+^ immobilized crosslinked PMAA layer coated Fe_3_O_4_ magnetic core, which was promising for practical applications of His-protein separation and purification in proteomics [82]. Yuan et al. developed superparamagnetic microspheres bearing phosphine oxide groups for effective extraction of uranium from highly acidic solution [83,84]. Chang et al. prepared Fe(II)-based coordination polymer nanohydrogels as a new type of nanozyme by RPP with *N*,*N*′-methylenebis(acrylamide) (MBA_m_) as a crosslinker, showing a regular spherical morphology with a larger size (Figure 4) [76].

### 2.2. Photoinitiated Precipitation Polymerization

Limé and Irgum prepared a series of highly cross-linked micrometer-sized polymer particles made from DVB, alone or copolymerized with 2,3-epoxypropyl methacrylate or styrene by irradiation from a 150 W short arc xenon lamp with AIBN as the initiator, and namely photoinitiated precipitation polymerization (PPP) [33,34]. By using photoinitiation instead of thermal initiation, it was possible to avoid coagulum and arrive at spherical particles with an exceptionally high monodispersity for particles of this size range (polydispersity index < 1.02) and with monomer loadings well above 5 % [85,86,87]. Moreover, the PPP method can be carried out at low temperatures with corresponding lower tendencies of aggregate formation and also allows the polymerization temperature and initiation rate to be varied independently [33]. Chemtob et al. summarized the main cross-linkers and monofunctional monomers used in PPP, as well as the polymerization mechanism that a transient polymer surface gel layer plays a key role in the colloidal stabilization of particles by a so-called “auto-steric” stabilization process [88].

Tugrul Cem Bicak first reported the combination of type II photoinitiation with PP for the synthesis of highly crosslinked and spherical PDVB particles with click functionality by using a benzophenone-tertiary amine initiation system [41]. Wang et al. prepared the composite of acrylamide on the surface of nano TiO_2_ under ultraviolet light by PPP, providing a facile strategy for the synthesis of organic-inorganic hybrid flocculants [89]. Zeng et al. prepared a new absorbent triethylene tetramine-modified crosslinked polyacrylonitrile in water at room temperature using FeC_2_O_4_ as the photoinitiator and MBA_m_ as a crosslinking agent, which could be used as an effective adsorbent for Cu(II) ions removal from wastewater [90].

### 2.3. Solvothermal Precipitation Polymerization

**Figure 5:**
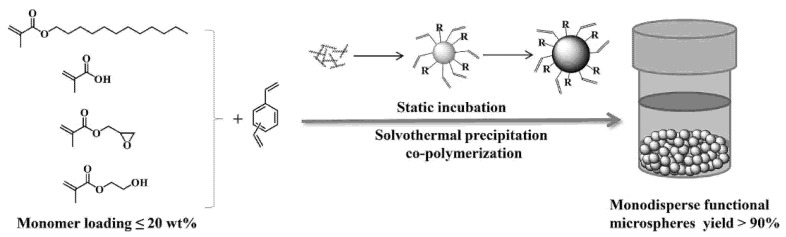
The synthetic scheme of the micro-sized monodisperse poly(methacrylic monomer-divinylbenzene) microspheres fabricated by solvothermal precipitation co-polymerization. Reprinted with permission from Ref. [92]. Copyright 2021 Elsevier B.V.

To address the challenges of high solid content and high microsphere yield in the fabrication of monodisperse microspheres (*ex*. the maximum monomer loading of DVB in ACN cannot exceed 5% vol., and the yield for common precipitation polymerization was always less than 50%), Chen et al. prepared monodisperse spherical PDVB, via solvothermal precipitation polymerization (SPP), by adding monomer, initiator, and solvent in a closed reaction vessel at temperatures above the boiling point of the solvent without stirring [35]. Due to its fascinating features, such as an enhanced reaction rate, high yields, uniformed size distribution, and the appropriate diameters ranging from nano-size to micron-size, the proposed SPP method could find numerous ways in the synthesis of a variety of monodisperse microspheres and the building of new inorganic–organic hybrid materials [91]. Chen et al. further prepared a series of micron-sized, highly crosslinked polymeric microspheres containing epoxy, lauryl, carboxyl, and hydroxyl groups by SPP at 20% (mass) monomer loading with over 94% microsphere yield (Figure 5), suggesting its potential value for large scale-up preparation of various monodisperse microspheres with a pure surface [92].

### 2.4. Controlled/‘‘Living’’ Radical Precipitation Polymerization

Living polymers with reactive end groups can be easily obtained via controlled/‘‘living’’ radical polymerization (CRP), which can be further extended to produce block polymers and polymers with other more complicated architectures. Zhang et al. first introduced the CRP mechanism into traditional polymer bead-forming, and proposed a series of controlled/‘‘living” radical precipitation polymerization (CRPP) approaches, including (normal and reverse) atom transfer radical precipitation polymerization (ATRPP), iniferter-induced ‘‘living’’ radical precipitation polymerization (ILRPP), and RAFT precipitation polymerization (RAFTPP) [37]. Other new techniques have also been developed, such as nitroxide-mediated precipitation polymerizations (NMPP) [93]. Similar to the TRPP, all CRPPs should involve the typical particle nucleation and growth stages, while their particle growth mechanisms are quite different. The particle growth mechanism of TRPP could be defined as “grafting to” the mechanism according to the grafting concept, while the normal ATRPP and ILRPP systems were suitable for “grafting from” particle growth mechanism, in which the polymer particles grow by directly capturing monomers from the reaction solutions through the surface-initiated CRPs [94,95]. In addition, a combined “grafting from” and “grafting to” particle growth mechanism was found to be present in the reverse ATRPP and RAFTPP systems, which could be attributed to the presence of both the “living” initiating or chain transfer groups on the polymer particles and traditional free radical initiators in the polymerization systems [96]. The Zhang group has presented detailed overviews of CRPP approaches and their advances in the preparation of advanced functional polymers [36,37,94], and Hasanah et al. recently published a review summarizing various CRPPs for preparing molecularly imprinted microspheres (MIMs) as well as comparing advantages and disadvantages of each technique [97]. In the following parts, we will summarize the related works in recent years.

#### 2.4.1. Atom Transfer Radical Precipitation Polymerization (ATRPP)

**Figure 6:**
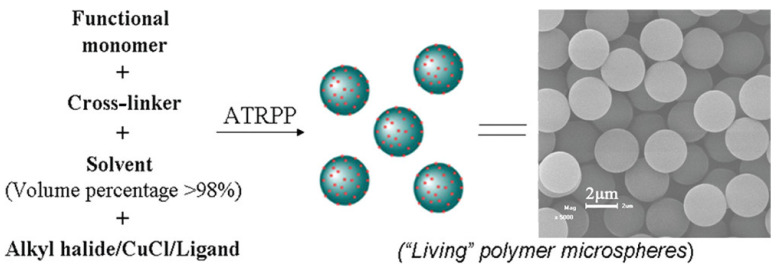
The synthetic scheme of the uniformly crosslinked “living” polymer microspheres. Reprinted with permission from Ref. [98]. Copyright 2011 American Chemical Society.

ATRPP is the incorporation of an ATRP system with a PP procedure, which is simply a substitute for the conventional initiator (e.g., AIBN) with an ATRP initiator. After activation under appropriate reaction conditions, all chains are quickly initiated and grow simultaneously, leading to soluble branched oligomers at the beginning of the polymerization. The controlled characteristics of ATRPP have major roles in uniform particle growth and size [98,99]. Efficient strategies have been continuously conducted to prepare high-quality polymer microspheres. Zhang et al. first introduced the ATRP mechanism into PP for the generation of uniformly “living” polymer microspheres with number-average diameters ranging from 0.73 to 3.25 μm and polydispersity indices being typically lower than 1.01 (Figure 6) [98]. Yang et al. combined the reverse ATRP mechanism with PP and obtained surface-functionalized “living” polymer microspheres via two-stage PP with better-controlled chain growth and size distribution, where the first stage is a conventional PP (for nucleation) and the second stage is a reverse ATRP (for particle growth) [100]. Cormack et al. first reported a new ATRP-based methodology, electron transfer atom transfer radical precipitation polymerization (ARGET ATRPP), in which the products are of high quality (in a size range of 1–3 μm) and can be used directly in grafting-from experiments without any need for the installation of initiator moieties [101].

#### 2.4.2. Iniferter-Induced ‘‘Living’’ Radical Precipitation Polymerization (ILRPP)

ILRPP is the most recently developed CRPP and functions by incorporating the ILRP system into PP by using an iniferter agent (as initiator, transfer agent, and the terminator at the same time during radical polymerization). The polymer particles in the ILRPP system mainly grow by directly capturing monomers (including both divinyl crosslinkers and monovinyl functional monomers) from the reaction solution through the surface-initiated controlled polymerization process. Similar to that in ATRPP, a ‘‘grafting from’’ growth mechanism should mainly work in ILRPP [95], where every part of the iniferters in ILRPP is immediately converted into macroiniferters at the onset of polymerization, though it showed less control when compared to ATRPP and RAFTPP. All the resulting polymer microspheres by ILRPP generally contained active iniferter groups on their surfaces, thus allowing further surface functionalization [102].

#### 2.4.3. RAFT Precipitation Polymerization (RAFTPP)

**Figure 7:**
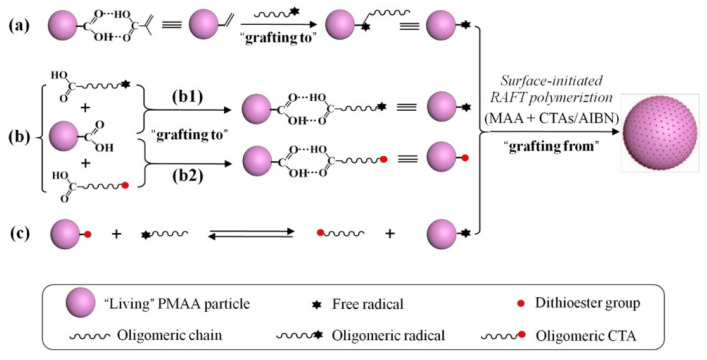
Three Proposed Particle Growth Mechanism in RAFTPP of MAA. (**a**) By capturing oligomeric radicals with particle surface vinyl groups; (**b**) By adsorbing oligomeric polymer chains from the continuous phase; (**c**) By the surface-initiated RAFT polymerization through the surface dithioester groups. Reprinted with permission from Ref. [48]. Copyright 2019 American Chemical Society.

RAFTPP was developed by introducing the RAFT polymerization mechanism into the PP system by simply adding an appropriate chain-transfer agent (or RAFT agent), which is highly promising for the development of various well-defined advanced functional polymer materials [103,104] used in drug delivery and molecular recognition (MIPs) [105,106]. Zhang et al. has developed a series of highly efficient approaches for the preparation of MIP particles with surface-grafted well-defined hydrophilic polymer layers, involving the “one-step approach” and “two-step approach” [107,108,109,110]. They have obtained “living” PMAA particles via RAFTPP of MAA or a mixture of MAA and a functional comonomer and expected that the growth of such “living” PMAA particles could take place not only by capturing oligomeric radicals with particle surface vinyl groups (Figure 7a) and adsorbing oligomeric polymer chains from the continuous phase through hydrogen-bonding interaction (Figure 7b) but also by the surface-initiated RAFT polymerization through the surface dithioester groups (Figure 7c) [48]. Subsequent surface-grafting polymerizations have also been obtained to prepare uniform hairy hollow polymer particles with different hydrophilic polymer brushes and hydrodynamic diameters [111].

Taken together, all the prepared advanced functional polymers possess great potential for applications such as controlled drug release vehicles, carriers for reagents, enzymes, catalysts, and so on. Other methods, such as free-radical precipitation polymerization [112], have also been developed rapidly and as a powerful support for biomedical applications.

### 2.5. Self-Stable Precipitation Polymerization

In recent years, the Yang group has developed a novel heterogeneous polymerization technique, termed self-stabilized precipitation polymerization (2SPP), beyond emulsion/dispersion/suspension. Compared with the TRPP method, this reaction system has the following characteristics: (1) High monomer concentration and the choice of the reaction medium is crucial; (2) Quiescent polymerization, a stable colloid composed of uniform polymer particles and reaction medium was formed through a self-nucleation and surface deposition process in the absence of any stabilizers; (3) The polymer particles can be easily separated from the solvent, and the supernatant liquid can be recycled, making this one of the most efficient, green and easily scaled-up strategies [113]. A possible mechanism was proposed for this 2SPP process that the nucleation of particles occurred homogeneously only at the early stage in the solution phase; polymer chains will aggregate and gradually grow and nucleate out of the solution. Then polymer chains are continuously deposited on the particles that were precipitated from the solution and eventually grow into balls [114]. Wang et al. further used a fluorescence self-imaging method based on aggregation-induced luminescence to monitor the process of particle generation and growth during 2SPP in a real-time and disturb-free manner [115].

A range of common olefinic monomers, including mono-olefins, di-olefins, and aromatic olefins, can undergo a 2SPP reaction in a specific solvent environment with maleic anhydride (MAH) or its derivatives. The copolymerization equipped with highly enhanced reactivity of MAH with vinyl acetate (VAc), styrene, α-methyl styrene, and DVB [116,117,118] has been achieved, and the obtained polymers could support further preparing bio-based, thermally stable, or fluorescent materials [119,120,121,122,123,124,125]. The 2SPP method also provides a new way to transform huge amounts of olefinic compounds in the C4~C9 fraction of the petrochemical industry and prepare polyolefin products containing acid anhydride functional groups. This convenient and efficient process can make up for deficiencies of the heterogeneous catalyst (Ziegler–Natta, Metallocenes, etc.) in the preparation of polar copolymers and getting well-defined nano- or micron-sized polymeric particles [38].

**Figure 8:**
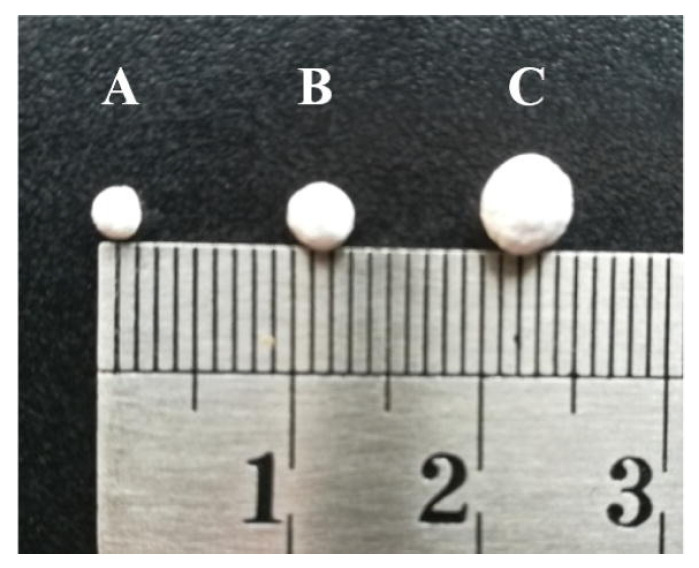
Images of typical polyethylene particles synthesized within 10–30 min (Polymerization time: (**A**) 10 min, (**B**) 20 min, (**C**) 30 min). Reprinted with permission from Ref. [125]. Copyright 2020 Elsevier B.V.

Recently, such heterogenization strategies have been explored in the field of ethylene polymerization [126] and ethylene–polar monomer copolymerization [127] to obtain polyolefin spherical particles with improved mechanical properties and great product morphology control. Through modulating polymerization parameters, the particles could be well-tuned, causing the precipitation and separation of polymers from reactive medium avoiding reactor fouling (see Figure 8). Chen et al. designed an ionic cluster strategy to synthesize polar-functionalized polyolefins via PP, which greatly enhances the catalyst’s thermal stability (90–150 °C) and enables the homopolymerization of both terminal and internal polar-functionalized olefins [127].

## 3. Conclusions and outlook

Precipitation polymerization is the most common method to prepare monodisperse polymer microspheres, which also allows further functionalization via the next process. Much effort has been made to precisely control the particle size of the internal pore structures to satisfy various application requirements. In addition to the approaches and applications described above, other new techniques have also been developed, making it an irreplaceable tool to prepare functional materials. For example, nitroxide-mediated PP employing photo-crosslinkable prepolymers [128]; catalyst-free aza-Michael addition PP method enabled polymer particles with abundant active groups for direct post-modification in mild conditions [129]; the aqueous free-radical PP method used to prepare thermo- or light-responsive responsive microgels [130,131,132]; water-based redox PP for synthesizing polypyrrole nanoparticles as a promising substance for photoacoustic imaging [133]. Novel particles have been creatively prepared, such as deoxyribonucleic acid (DNA) cross-linked polymeric nanoframework [134] or those supporting Pd or Au catalysts [135,136], exhibiting great potential in both material science and bio-medical areas.

The improved PP method resulted in microspheres with controlled particle size and active groups on the surface; the amount of monomer input and the yield of the particles were increased with guaranteed microsphere monodispersity; thus, providing strong support for the next step of industrial scale-up production and fitting specific applications in various areas. In the future, the introduction of each advanced technique into the area of precipitation polymerization is expected to progress and achieve great product morphology control and precise application rapidly.

Precipitation polymerization is a powerful tool for the preparation of uniform polymer particles with a clean surface and can also be easily combined with other techniques. How to further reduce the production cost, simplify the production process, and expand the production scale will also be the focus of much attention and research in the future.

## Data Availability

Not applicable.
